# Medical mud-pack treatment with different temperatures in patients with knee osteoarthritis

**DOI:** 10.1007/s00484-025-02864-0

**Published:** 2025-02-10

**Authors:** Fulya Demircioğlu Güneri, Fatih Karaarslan, Hülya Özen, Ersin Odabaşi

**Affiliations:** 1 Department of Medical Ecology and Hydroclimatology, Gülhane Faculty of Medicine, University of Health Sciences, Ankara, Turkey; 2 Department of Medical Informatics, Gülhane Faculty of Medicine, University of Health Sciences, Ankara, Turkey

**Keywords:** Knee osteoarthritis, Medical mud-pack treatment, Thermal therapy, Balneotherapy, Peloidotherapy

## Abstract

To compare the effects of medical mud-pack (MMP) treatments applied at different temperatures on the pain and joint functions of patients with knee osteoarthritis (KOA). Kellgren Lawrence (KL) stage 3 or 4 KOA patients were included and randomized into three groups. Patients in groups 1, 2, and 3 took MMP treatment to both knees at 39 °C, 42 °C, and 45 °C, respectively. The treatment was performed for 12 days (only weekdays) and was 30 min long per day. The same blinded physician evaluated the patients at baseline and at the end of the treatment. The assessments were done before and after the intervention. The primary outcome was to achieve a minimal clinically important improvement (MCII) for KOA (decrease of at least 19 mm (-40.8%) on the VAS for pain, a decrease of 18.3 mm (-39%) on the patient’s global assessment (PGA), and/or a decrease of at least 9.1 points (-26%) on the Western Ontario and McMaster Universities Osteoarthritis Index function subscale (WOMAC-FS). Secondary outcomes were pain (VAS), patient’s global assessment (VAS), physician’s global assessment (VAS), Western Ontario and McMaster Universities Osteoarthritis Index (WOMAC), Patient’s health state, Patient Acceptable Symptom State (PASS). 217 patients were analyzed. Groups 1, 2, and 3 had 68, 81,68 patients, respectively. The MCII measurement revealed that MMP treatment did not show a significant difference between groups 2 and 3 (*p* > 0.05). Also, it was observed that more patients in groups 2 and 3 reached the MCII compared to group 1 (*p* < 0.001). For the secondary outcomes, significant improvements were observed within-group evaluations for each of the three groups (*p* < 0.001). Between groups comparisons, the improvements at the end of the treatment were found to be superior for group 2 and group 3 compared to group 1 (*p* < 0.001). There was no statistically significant difference between groups 2 and 3 for any parameters (*p* > 0.05). The number of patients who achieved the PASS was statistically lower for group 1 compared to groups 2 and 3 (*p* < 0.001). We observed significant improvements in all groups after treatment. The main result, as measured by MCII, suggests that MMP treatments at 42–45 °C is more effective than at 39 °C in managing severe KOA patients’ pain and functional status. We found no significant difference in pain and joint function improvement between 42 °C and 45 °C after MMP.

## Introduction

Osteoarthritis is a prevalent form of arthritis that affects around 302 million people worldwide. It is the primary cause of disability among the elderly population (Kolasinski et al. 2021). The most common type of osteoarthritis occurs in the knee. Cartilage degradation, bone remodeling, osteophyte formation, and synovial inflammation are osteoarthritis’s most common pathological findings. Patients typically experience symptoms such as pain, stiffness, swollen joints, and movement disability. Treatment usually involves a combination of medical and non-medical interventions (Michael et al. 2010).

Medical muds (peloids) are substances that can be either organic or inorganic compounds. They have been used for decades to apply heat to deep tissues. These substances are commonly administered regionally through packs or baths heated to appropriate temperatures. According to literature, medical mud-packs (MMP) are usually used at temperatures ranging from 39 to 45 °C for 10–20 min over 10–15 days. Medical mud-pack treatment is a non-invasive method that is easy to use, has a low risk of adverse effects, and is well-tolerated by patients (Forestier et al. 2017).

The Osteoarthritis Research Society International (OARSI) guidelines 2014 recommended balneotherapy, which includes various thermal interventions, as a suitable non-surgical treatment option for knee osteoarthritis (KOA) patients with high co-morbidities and multiple joint involvement (McAlindon et al. 2014). Furthermore, The American College of Rheumatology (ACR) has suggested various thermal applications for managing knee, hip, and hand osteoarthritis (Kolasinski et al. 2021).

In the literature, several studies have shown that MMP treatment, as part of balneotherapy, can reduce pain and improve functional status, enhancing the quality of life for patients with KOA (Fraioli et al. 2018; Forestier et al. 2016). However, there is a lack of standardized application methods, including parameters such as application temperatures and duration for MMP treatment. Since MMPs treatments are frequently used to treat KOA, we think that the optimal treatment methods should be studied for these patients. So, we conducted a study to compare the effects of MMP applied at different temperatures on the pain and joint functions of patients with KOA. Our findings reveal the optimal application temperatures for MMP and emphasize the importance of standardization. This study can serve as a basis for further research on this topic.

## Material methods

### Trial design

This study is a randomized, parallel, 1:1, controlled, double-blind, prospective trial that compares the effects of MMP treatment administered at three different temperatures on the pain and functional status of patients with KOA.

### Ethics Statement

The protocol (No:941) was approved by the Ankara City Hospital Number 1 Clinical Researches Ethical Committee on 16/07/2020.

### Participants

#### Inclusion criteria

Our study included patients between the ages of (45–80) who were diagnosed with primary KOA according to the American College of Rheumatology (ACR) diagnosing criteria (Altman et al. 1986) and had grade 3–4 KOA according to Kellgren Lawrence criteria (Kellgren and Lawrence 1957). Additionally, they had to have a minimum of 40 visual analog scores (VAS) in pain assessment and be willing to sign the written consent form.

#### Exclusion criteria

Patients with skin or systemic conditions labeled contraindicated for local heat applications, intra-articular injection, electrotherapy, balneotherapy, acupuncture treatments in the previous six months, and knee trauma or surgery during the last six months were excluded from our study.

### Settings

The study was conducted in the MMP treatment unit of the Medical Ecology and Hydroclimatology Department at Health Sciences University, Gülhane Education and Research Hospital between 20/07/2020 and 1/07/2024.

### Intervention

We used medical mud from the Muğla Region in Turkey, which contain a high level of humic acid as well as organic and inorganic components (Karaarslan et al. 2021). This medical mud has been analyzed by licensed public health department laboratories. No unauthorized chemical or microbiological contaminants were found.

We categorized our patients into three groups to receive MMP treatment at different temperatures. Group 1 was treated at 39 °C, Group 2 at 43 °C, and Group 3 at 45 °C. The treatment involved three steps. First, a 0.5 cm layer of mud at the desired temperature was applied directly to the skin of the patient’s knees. Next, the patient was covered with stretch film over the mud layer, followed by placing an external mud pack, also at the same temperature, on top of the stretch film. This external mud pack was prepared in a 25 × 45 × 3 cm nylon cover. The nylon-covered mud packs were prepared to the required temperature using the bain-marie method. They utilized a digital thermometer to measure the temperature of the medical mud and applied the treatment as soon as the desired temperature was reached. Finally, after fifteen minutes, the mud packs were replaced with new ones at the initial temperature. The entire procedure was carried out by skilled and experienced nurses. Each treatment session lasted for 30 min, and the regimen consisted of twelve consecutive days of sessions, excluding weekends.

The patients in both groups were instructed to continue taking their medications for their systemic diseases. During the session, we observed the need for analgesic drugs. The supervisor physician responsible for the routine functioning of the treatment unit checked daily to ensure that healthcare providers followed the protocol and that patients were compliant. A pre-set alarm clock was used to maintain timing.

### Outcomes

The same physician (FDG) evaluated the patients at baseline and at the end of their treatment. Demographic details such as age, sex, height, weight, body mass index (BMI), and disease-related information such as the duration of symptoms and routine medical treatment (non-steroidal anti-inflammatory drugs (NSAID) or pain killers) were recorded at the time of inclusion.

#### Primary outcome

The main goal of the study was to achieve a minimal clinically important improvement (MCII) in VAS for pain, patient’s global assessment, and WOMAC function subscale for KOA which is previously defined by Tubach et al. (2005a). These improvements were a decrease of at least 19 mm (-40.8%) on the VAS for pain, a reduction of 18.3 mm (-39%) on the patient’s global assessment, and/or a decrease of at least 9.1 points (-26%) on the WOMAC function subscale.

#### Secondary outcomes

1-Pain intensity- Visuel Analog Scale (VAS) (0–100 mm)

We used the VAS ranging from 0 to 100 mm to evaluate the patient’s pain intensity. The patients were asked to indicate the level of their pain on the scale by marking the point that represents their pain level. The scale ranges from 0 (no pain) to 100 (unbearable pain). This assessment method has been previously established by Huskisson (1974), Million et al. (1982) and Gallagher et al. (2001).

2-Western Ontorio and McMaster Universities Osteoarthritis Index (WOMAC)

We used the five-scale Likert of WOMAC and obtained the total score by summing the scores of pain, stiffness, and physical functioning subgroups. The Turkish language validation study of WOMAC was conducted by Tüzün EH. (Tüzün et al. 2005).

3-Patients’ global assessment- Visual Analog Scale (VAS) (0–100 mm)

The patients’ overall evaluations were conducted using VAS ranging from 0 to 100 millimeters.

4-Patients’s health state

We surveyed the patients to assess their symptoms using a 5-point Likert scale before and after the treatment. The scale ranged from one to five, with the options being “very bad,” “bad,” “moderate,” “better,” and “much better.”

5-Patient Acceptable Symptom State” (PASS)

We conducted research on the number of patients who have reached the Patient Acceptable Symptom State (PASS). To determine the acceptable symptom state, we asked the patients the following question: “When you review all the activities that you have in your daily life, do you find your final status satisfactory?” They could answer yes or no to this question. This concept helps us evaluate the well-being of patients by understanding their current symptom status that they consider acceptable. Even if the scores improve at the end of the treatment, some patients may still find the improvement inadequate, especially those with high pain scores at the beginning. PASS allows us to determine better patients’ satisfaction levels (Tubach et al. 2005b).

### Randomization and assignment

We utilized a table of computer-generated random numbers for randomization. The researcher, blinded to the treatment assignments, randomly allocated the patients to their respective groups and provided instructions for completing the self-assessment questionnaires. The patients were assessed on the first and last day of their treatment sessions. To prevent interaction between the patients in different groups, they were taken to separate rooms for their treatment sessions.

### Blinding

Our study employed a double-blind design to ensure unbiased results. In this approach, neither the physicians performing follow-up evaluations nor the patients were aware of the MMPs’ temperatures in the treatment. Only the nurses responsible for administering the treatments knew the specific temperature each patient received. These nurses were instructed to keep this information confidential from the patients and the physicians. Treatments were scheduled at different times throughout the day to minimize patient interactions. Additionally, an independent statistician, who was unaware of the group assignments, conducted all statistical analyses to maintain objectivity in the study’s findings.

### Statistical analysis

A priori power analysis was conducted to determine the number of patients necessary for inclusion in the study groups. The analysis focused on comparing the quantitative outcomes measured across the three groups. With a power of 80%, a type I error rate of 5%, and a medium effect size (f = 0.25), it was calculated that at least 53 participants per group were required for the study. Since repeated measures would be performed throughout the study, the aim was to include at least 64 patients per group to account for potential missing data and ensure that the data analysis remains robust. We used the IBM SPSS 25 program for data analysis. For quantitative variables, we presented descriptive statistics as mean ± standard deviation or median (Q1-Q3 for non-normally distributed data), and for qualitative variables, we used frequency and percentage. We assessed normality of quantitative variables using the Shapiro-Wilk test. We used one-way analysis of variance (ANOVA) for normally distributed data and the Kruskal-Wallis test for non-normally distributed data to compare independent groups over quantitative variables. Pairwise comparisons were done using Dunn’s test after significant results from the Kruskal-Wallis test. To assess the relationship between qualitative variables, we conducted Pearson chi-square analysis. Quantitative outcomes measured at the beginning and end of the study were assessed using two-way mixed ANOVA with general linear models for repeated measures. The pairwise deletion method was used to address missing data when constructing the ANOVA models. Each model included group and time as main effects and group * time as the interaction effect. Post hoc testing was performed only for significant interactions, using a simple effect analysis with Bonferroni adjustment. Summary statistics of these models were presented as mean ± standard error of the mean. We considered p-values less than 0.05 as significant.

## Results

### Participant Flow

**Fig. 1:**
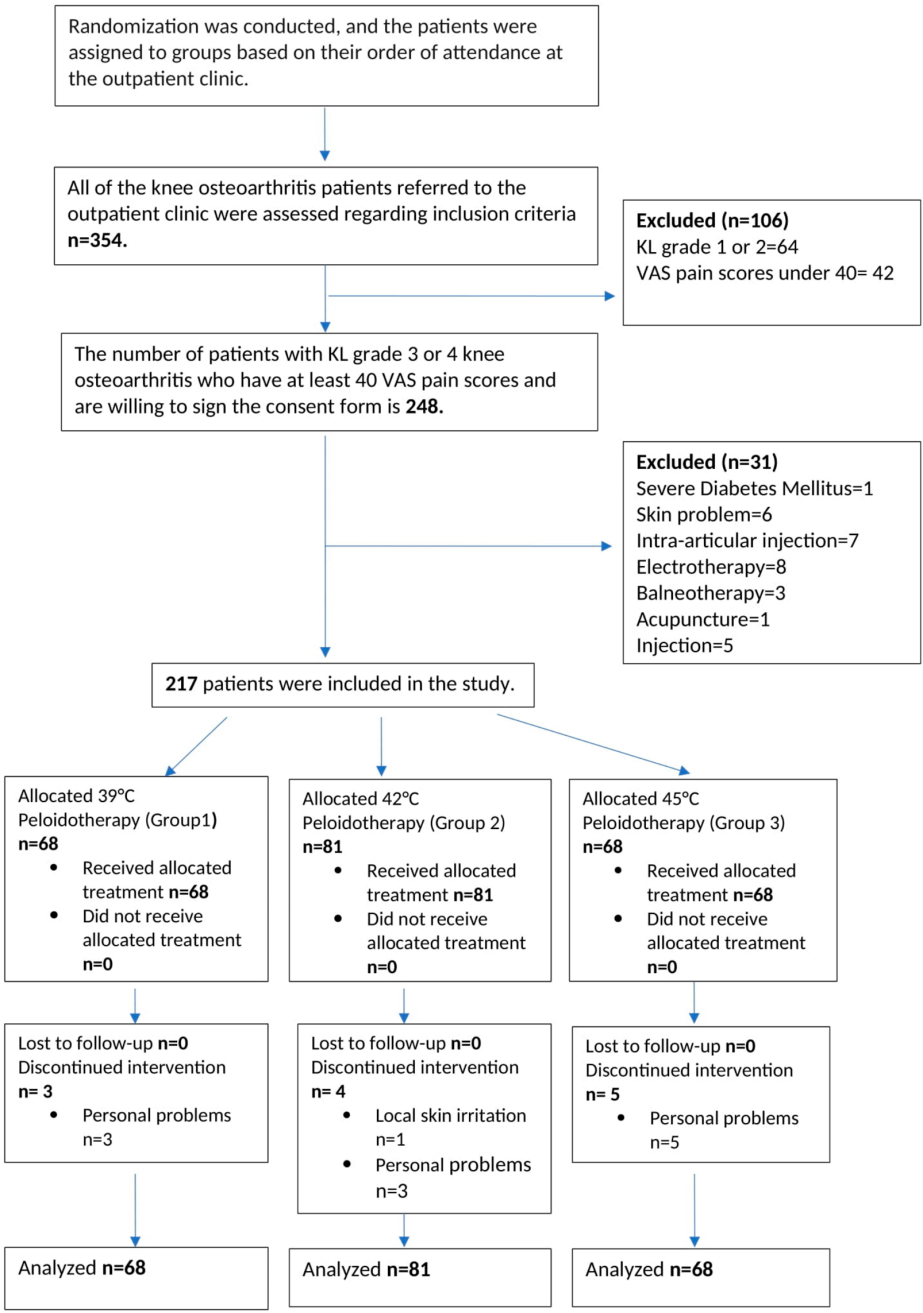
Flow diagram

We presented the flow diagram in Fig. 1.

### Recruitment

We assessed 354 KOA patients who visited our outpatient clinic between 20/07/2020 and 01/07/2024. During this period, we identified 248 KOA patients with KL grade 3 or 4 and VAS pain scores of 40 and above willing to provide written informed consent. Thirty-one of them were excluded, leaving 217 patients enrolled in the study.

### Losses and exclusions

We completed the initial evaluation for all patients in three groups. In group 1, 3 patients; in group 2, 4 patients; and group 3, 5 patients did not continue the sessions. The most common reason cited was personal, workplace, or family problems. Only one patient in group 2 had to discontinue due to sensitive skin and irritation after the third application.

### Number analyzed

217 patients were included in the intention-to-treat analysis for primary and secondary outcomes.

Baseline data.

We have summarized the patients’ baseline characteristics in Table 1. Except for the body mass index parameter, all patient characteristics were similar between the groups. We found that patients’ body mass index in group 2 was statistically higher than in the other groups (*p* = 0.028).

**Table 1:** The baseline characteristics of the patients

Variables		Group 1 (39 °C) (*n* = 68)	Group 2 (42 °C) (*n* = 81)	Group 3 (45 °C) (*n* = 68)	*P*
Age		62.31 ± 9.11	59.02 ± 8.59	61.12 ± 8.10	0.063*
Gender	Female Male	64(94.1%) 4(5.9%)	70(86.4%) 11(13.6%)	55(80.9%) 13(19.1%)	0.069**
Body mass index (kg/m2)		29.62(26.37–32.74)^a^	31.02(28.23–35.38)^b^	28.91(26.73–32.84)^a^	0.028***
Analgesic consumption	Yes No	29(42.6%) 39(57.4%)	34(42%) 47(58%)	31(45.6%) 37(54.4%)	0.898**
Radiologic state (KL scale)	Grade 3 Grade 4	41(60.3%) 27(39.7%)	58(71.6%) 23(28.4%)	43(63.2%) 25(36.8%)	0.318***
VAS Pain (0–100 mm)		75(57.5–80)	78(60–88)	80(60–90)	0.248***
VAS PGA		70(53.5–80)	70(50–80)	70(51–80)	0.855***
VAS PhGA		75(60–80)	70(60–80)	77.5(60-82.5)	0.704***
WOMAC TS		52.31 ± 16.01	48.81 ± 15.75	51.16 ± 16.63	0.400*
WOMAC FSS		54.66 ± 17.88	50.68 ± 17.41	52.68 ± 17.54	0.390*
Patient status (1–5 Likert Scale)		5(4–5)	5(4–5)	5(4–5)	0.243***

Bold value indicates significant result (*p* < 0.05)

* One-way ANOVA

** Pearson Chi-square test

*** Kruskal-Wallis H test

PGA Patient’s Global Assessment, PhGA Physician’s Global Assessment, WOMAC: Western Ontario and McMaster Universities multifunctional index, WOMAC TS: WOMAC total score, WOMAC FSS: WOMAC function subscale score

### Outcomes

#### Primary outcome

According to the MCII measurement, the primary finding indicates that MMP treatment did not significantly differ between groups 2 and 3 (*p* > 0.05). However, it was observed that significantly more patients in groups 2 and 3 reached the MCII compared to group 1 (*p* < 0.001). Table 2 provides a breakdown of the number of patients who achieved minimal clinically important improvement.

**Table 2:** Primary outcome - number of patients with minimal clinically important improvement (MCII) at the end of the treatment and inter-group comparison

Variables	Group 1 (39 °C) *n* = 65	Group 2 (42 °C) *n* = 77	Group 3 (45 °C) *n* = 63	*P*
VAS Pain (≥ 40.8%)†	3 (4.6%)^a^	36 (46.8%)^b^	37 (58.7%)^b^	< 0.001*
VAS PGA (≥ 39%)†	6 (9.2%)^a^	32(41.6%)^b^	37 (58.7%)^b^	< 0.001*
WOMAC FSS (≥ 26%)†	11 (16.9%)^a^	37 (48.1%)^b^	31 (49.2%)^b^	< 0.001*

^a, b^: There is no significant difference between column percentages with the same letter in the same row (*p* > 0.05); bold values indicate significant results

*Pearson Chi-square test

PGA Patient’s Global Assessment, WOMAC: Western Ontario and McMaster Universities multifunctional index, WOMAC FSS: WOMAC function subscale score

†:(MCII): VAS Pain (≥ 40.8%), VAS PGA (≥ 39%), WOMAC FSS (≥ 26%)

#### Secondary outcomes

We have presented the secondary outcomes in Table 3.

**Table 3:** Results of secondary outcomes

Variables	Time	Groups			P*			Between group comparisons		
		Group 1 (39 °C)	Group 2 (42 °C)	Group 3 (45 °C)	Time	Group	Time*Group	(1–2)	(1–3)	(2–3)
VAS Pain (0–100 mm)	Baseline	70.34 ± 2.03	73.07 ± 2.05	75.07 ± 1.97	**< 0.001**	**0.008**	**< 0.001**	0.903 < 0.001**	0.332 < 0.001**	1.000 0.339**
	Post treatment	49.17 ± 2.42	32.79 ± 2.21	27.59 ± 2.36						
	Within Group comparisons	< 0.001**	< 0.001**	< 0.001**						
VAS PGA	Baseline	67.22 ± 2.01	67.8 ± 1.84	68.88 ± 1.96	**< 0.001**	**0.001**	**< 0.001**	1.000 < 0.001**	1.000 < 0.001**	1.000 0.542**
	Post-treatment	48.68 ± 2.23	31.94 ± 2.13	27.71 ± 2.37						
	Within Group comparisons	< 0.001**	< 0.001**	< 0.001**						
VAS PhGA	Baseline	70.71 ± 2.01	70.4 ± 1.86	72.71 ± 1.8	**< 0.001**	**< 0.001**	**< 0.001**	1.000 < 0.001**	1.000 < 0.001**	1.000 0.340*
	Post-treatment	48.85 ± 2.35	31.95 ± 2.18	27.05 ± 2.01						
	Within Group comparisons	< 0.001**	**< 0.001**	< 0.001**						
WOMAC TS	Baseline	52.31 ± 1.94	48.81 ± 1.75	51.16 ± 2.02	**< 0.001**	**0.001**	**< 0.001**	0.717 < 0.001**	1.000 < 0.001**	1.000 1.000*
	Post-treatment	36.6 ± 2.1	22.68 ± 1.53	23.38 ± 1.64						
	Within Group comparisons	< 0.001**	< 0.001**	< 0.001**						
WOMAC FSS	Baseline	54.66 ± 2.17a	50.68 ± 1.93	52.68 ± 2.13	**< 0.001**	**0.001**	**< 0.001**	0.689 < 0.001**	1.000 < 0.001**	1.000 1.000*
	Post-treatment	39.29 ± 2.42	23.75 ± 1.68	24.25 ± 1.75						
	Within Group comparisons	< 0.001**	< 0.001**	< 0.001**						
Patient status (1–5 Likert Scale)	Baseline	4.59 ± 0.07	4.56 ± 0.06	4.69 ± 0.06	**< 0.001**	**< 0.001**	**< 0.001**	1.000 < 0.001**	0.985 < 0.001**	0.465 0.218**
	Post-treatment	3.68 ± 0.12	2.79 ± 0.12	2.48 ± 0.14						
	Within Group comparisons	< 0.001**	< 0.001**	< 0.001**						
Number of patients who have achieved PASS		43 (66.2%)^a^	74 (96.1%)^b^	61(96.8%)^b^	< 0.001***					

Bold values indicate significant results (*p* < 0.05),*:Two way Mixed ANOVA, **: Simple effect analysis with Bonferroni adjustment (Bonferroni test), ***:Pearson Chi-square analysis, ^a, b^: different letters in the same row show statistical significance (*p* < 0.05), PGA Patient’s Global Assessment, PhGA Physician’s Global Assessment, WOMAC: Western Ontario and McMaster Universities multifunctional index, WOMAC TS: WOMAC total score, WOMAC FSS: WOMAC function subscale score, PASS: Patient acceptable symptom state

For all of the outcomes, we observed significant improvements within group evaluations for each of the three groups (*p* < 0.001). On the other hand, when comparing between groups, the improvements at the end of the treatment were found to be superior for group 2 and group 3 compared to group 1 (*p* < 0.001). There was no statistically significant difference between groups 2 and 3 for any of the parameters (*p* > 0.05). Additionally, the number of patients who achieved the PASS was statistically lower for group 1 compared to groups 2 and 3 (*p* < 0.001).

### Side effects

We monitored the patients for potential side effects during their treatments. Only one patient in group 2 experienced a skin reaction (redness and mild swelling) on the third day of treatment, prompting us to discontinue their treatment. No additional side effects were observed in any of the other treatment groups.

## Discussion

In our study, we observed significant improvements in all groups after treatment. The main result, as measured by MCII, suggests that MMP treatment at 42–45 °C is more effective than at 39 °C in managing the pain and functional status of severe KOA patients. Importantly, we found no significant difference in pain and joint function improvement between 42 °C and 45 °C after MMP treatment. To the best of our knowledge, our study is the first in the literature to compare the effects of MMP applications at different temperatures for KOA treatment. Reviewing the literature, we found that there is no standard temperature for MMP applications in KOA. Studies focusing on the effects of MMP treatment on pain and functional status for KOA patients have reported mud-pack temperatures ranging from 36 °C to 50 °C (Varzaityte et al. 2020; Benini et al. 2021). However, the most commonly used temperatures for the treatment are 42 °C (Kiraly et al. 2020; Tefner et al. 2013; Evcik et al. 2007; Fioravanti et al. 2015) and 45 °C (Güngen et al. 2012; Forestier et al. 2010; Özkuk et al. 2017; Cantarini et al. 2007; Fioravanti et al. 2011; Odabasi et al. 2009; Bostan et al. 2010). Additionally, the trials which performed at rare temperatures like 39 °C (Flusser et al. 2002; Gouvêa et al. 2021), 43 °C (Odabasi et al. 2008; Adıgüzel et al. 2022), and 47 °C (Peluso et al. 2016) have also been studied. While some of these studies only involve MMP (Kiraly et al. 2020; Tefner et al. 2013; Evcik et al. 2007; Güngen et al. 2012; Odabasi et al. 2009; Bostan et al. 2010; Gouvêa et al. 2021; Odabasi et al. 2008), others combine thermal water bath treatments as well (Fioravanti et al. 2015; Fraioli et al. 2011; Vaht 2008; Benini 2021 Adıgüzel 2022). Using local MMP in combination with a thermal bath can affect the body’s heat load differently. When combined with a thermal bath, MMP applications can have a more widespread impact on the body’s temperature and may trigger more robust heat-related responses (Odabasi and Turan 2022). Since such an effect is not expected in regional MMP applications, delivering maximum heat to the knee area may be important. We used a different method compared to the studies mentioned above. The typical method involves spreading hot medical mud on the skin and covering it with a cloth. However, we applied the mud to the skin and then applied heated mud-packs. During the treatment, we used another mud-pack with the initial temperature to better maintain the desired treatment temperature. Therefore, the partial improvements we obtained at 39 °C may be related to this difference in our application method. This indicates that the applied temperature stability of MMP is crucial.

On the other hand, the “the hotter is the better” option wouldn’t be valid in our scenario since our study showed no statistically significant difference between the 42 °C and 45 °C. Even though various theories about the mechanisms of action could be proposed, it is difficult to interpret the results, which show no significant difference between the two temperatures. These theories are insufficient to fully understand heat’s indirect and complex effects on the human body (Cramer et al. 2022; Gàlvez et al. 2018). One of the emerging topics in the literature explaining the impact of heat is Transient Receptor Potential Vanilloid 1 (TRPV1), which is related to heat transmission, pain, and the inflammatory process. Heat stimuli and capsaicin are among the ligands of these receptors. It is noted that activation occurs at temperatures above 42 °C, the threshold for TRPV1. So, the results of our study for groups 2 and 3 may be related to the fact that TRPV1 receptors are activated by both mud-packs prepared at 42 °C and 45 °C (Liao et al. 2024; Bevan et al. 2014). In addition, the relationship between application temperature and the effects may plateau after a linear increase, similar to the relationship between heat and vasodilation (Johnson and Kellogg 2010). Still, we can’t be confident about the extent of these mechanism’s roles in the overall effect.

In addition, since we derive all the benefits from applications below 45 °C, lower heat exposure may be beneficial in some cases, especially for conditions like diabetes mellitus, where heat perception may be impaired. High heat exposure for these patients can lead to undesirable side effects such as burn injuries (Petrofsky et al. 2008; Sene 2018). It has been reported that even patients with no accompanying diseases experience pain perception at temperatures over 43 °C. Burn injury starts when the basal epidermis reaches 44 °C, and the damage rate increases logarithmically with temperature (Martin 2017).

### Limitation

One of the study’s limitations is that the assessment is based on self-assessment questionnaires, so patients may manipulate the answers as they wish. The patients in our trial were not informed about the temperatures used during the MMP treatments. To minimize communication and comparisons among patients, we scheduled their treatments at different times. Additionally, individual sensitivity to heat varies, causing perceptions of temperature to differ from person to person. As a result, no patient could definitively know the exact temperature of their treatment. However, some patients may have inferred the temperature used for their MMP treatments. Those who believed they were assigned to the 39 °C group may have been influenced by this assumption, which could have contributed to lower scores on the self-assessment questionnaire.

Finally, there is no control group for which we did not give any treatment, which decreases our study’s power.

## Conclusion

With this trial, we revealed that MMP treatment at 42 °C and 45 °C is more effective than 39 °C in relieving pain and improving knee joint function for patients with KOA. Since there is no difference in efficiency, we suggest using 42 °C MMP over 45 °C to protect the skin from unnecessary stress. More trials should be performed to enhance our results.

## Data Availability

Data sharing is not applicable to this article as no new data were created in this study.
